# Macrophages at the Crossroad of Meta-Inflammation and Inflammaging

**DOI:** 10.3390/genes13112074

**Published:** 2022-11-09

**Authors:** Lili Qu, Alyssa J. Matz, Keaton Karlinsey, Ziming Cao, Anthony T. Vella, Beiyan Zhou

**Affiliations:** 1 Department of Immunology, School of Medicine, University of Connecticut, Farmington, CT 06030, USA; 2 Institute for Systems Genomics, University of Connecticut, Farmington, CT 06030, USA

**Keywords:** macrophage, obesity, aging, meta-inflammation, inflammaging

## Abstract

Macrophages are central players in systemic inflammation associated with obesity and aging, termed meta-inflammation and inflammaging. Activities of macrophages elicited by the two chronic conditions display shared and distinct patterns mechanistically, resulting in multifaceted actions for their pathogenic roles. Drastically expanded tissue macrophage populations under obesity and aging stress attribute to both enhanced recruitment and local expansion. Importantly, molecular networks governing the multifaceted actions of macrophages are directly altered by environmental cues and subsequently contribute to metabolic reprogramming, resulting in meta-inflammation in obesity or inflammaging in aging. In this review, we will summarize how meta-inflammation and inflammaging affect macrophages and the molecular mechanisms involved in these processes.

## 1. Introduction

With the advancement of medical technology, the average life span worldwide has increased significantly, especially in developed countries. Based on the report from World Population Ageing 2017 Highlights, the number of people aged 60 years and older will reach 2.1 billion by 2050 [1]. This population shift will be accompanied by increased strain on health care systems due to the physiological and pathological conditions associated with aging. Aging is a progressive, multifactorial process, characterized by functional deterioration at the organ, tissue, cellular, and molecular levels. A state of chronic, low-grade systemic inflammation during aging is termed inflammaging, and is associated with aging-induced health risk [2]. During the last few decades, mounting evidence suggests a central role for macrophage malfunction in inflammaging development.

In addition to the expanding elderly population, the prevalence of obesity is rising. Obesity is the fifth leading cause of death worldwide and by 2030, 57.8% of the global adult population is projected to be overweight or obese [3]. Obesity-driven metabolic dysfunction and chronic inflammation, termed meta-inflammation, can progress toward serious conditions such as type II diabetes mellitus (T2D), non-alcoholic fatty liver disease (NAFLD) [4], cardiovascular disease, stroke, and cancers [5,6,7,8]. Studies revealed that higher BMI during young adulthood and middle age significantly increases the chance of hospitalization and mortality after 65 years of age [9]. Fat accumulation in the trunk and visceral areas, which is common in elderly adults, displays a greater health hazard than on hips and limbs, attributed to enhanced inflammatory features associated with these fat depots and phenotypically resemble cellular signatures revealed in obesity [9,10,11]. Further investigation into the association between meta-inflammation and inflammaging is necessary to improve mitigation strategies for age- and obesity-related health risks.

Macrophages are the most common immune population in almost all tissues and act as major players to ensure the homeostatic function of their host tissues under normal physiological conditions. Meanwhile, the plasticity of macrophages also allows them to respond to acute or chronic cues with swift, fine-tuned, and diverse responses, executing crucial functions during pathogen invasion or modulating chronic stress, such as in obesity and aging [12]. Obese and elderly individuals both have higher levels of circulating inflammatory markers, indicating chronic inflammation [13]. Macrophages, as primary mediators of inflammation in the circulation and tissues, act as a “bridge” to connect obesity and aging. In this review, we will summarize current understandings of macrophage actions in response to meta-inflammation and inflammaging and provide information for future exploration in the field to guide mitigating strategy development to improve the quality of life for obese and elderly populations.

## 2. Meta-Inflammation and Its Link to Inflammaging

Meta-inflammation describes the systemic chronic low-degree inflammation incurred during weight gain and perpetuated during obesity. Whereas inflammaging refers to the increased basal immune activation, juxtaposed with declining immune response in aged individuals. Clinical manifestations of meta-inflammation and inflammaging share many similar phenotypes: both feature elevated levels of inflammatory markers, including C-reactive protein (CRP), interleukin-6 (IL-6), and tumor necrosis factor-α (TNF-α). Their etiologies are also characterized with metabolic dysfunction, inherent to both obesity and aging. The chronic, systemic inflammation entailed by meta-inflammation in youth and middle-aged adults accelerates the onset of declining immune function in inflammaging. To better understand the link between meta-inflammation and inflammaging, it is necessary to understand their unique and common molecular features and functional impacts.

### 2.1. Meta-Inflammation

Several compelling factors have been posited for the development of obesity-induced inflammation; however, these factors are not comprehensive, and elaboration is needed. Fat accumulation is the direct result from imbalanced energy uptake and consumption, including caloric intake exceeding calorie expenditure, attributed to diet, lack of physical activity, and/or endocrine changes that disturb nutrient utilization and hinder metabolic rate. Expanded fat tissues experience hypoxia-associated cellular stress increase, leading to exhausted adipocyte cell death, which, in turn, upregulate chemokine pro-inflammatory molecule production, as well as dysregulated adipokines. As fat is amassed, adipocytes in the trunk and visceral adipose tissue (VAT) become hypertrophied with an increased turnover rate. This tumultuous, pro-inflammatory tissue environment activates local immune cells, including macrophages, and increases the recruitment of monocytes and other leukocytes into the VAT [14,15]. Obesity is also marked by an accumulation of circulating danger-associated molecular patterns (DAMPs), such as free fatty acids, cholesterol crystals, and extracellular nucleic acids from dying cells and increased intestinal permeability, exposing the body to increased pathogen-associated molecular patterns (PAMPs), such as lipopolysaccharide (LPS) [16,17]. These DAMPs and PAMPs can activate immune responses through Toll-like receptors (TLR) and NLR family receptors on/in immune and non-immune cells. Immune cell activation and infiltration into VAT contribute further to the high levels of inflammatory markers observed in meta-inflammation, both perpetuating inflammation and directly impacting cellular metabolism [17].

Obesity typically incurs an unhealthy increase in plasma glucose levels due to insulin resistance (IR). Insulin resistance affects insulin-sensitive tissues (skeletal muscle, liver, and adipose tissue), evoking less responsiveness to insulin action, and resulting in impaired glucose uptake, glycogen synthesis, and glucose utilization for energy production. Meta-inflammation-associated cytokines can independently trigger IR through activation of the JNK and NF-κB pathways in myeloid and insulin-targeted cells. JNK activation directly hinders insulin signaling through the inhibition of insulin receptor substrate-1 (IRS-1), a mediator of insulin’s functions towards stimulating glucose uptake, glycogen synthesis, and inhibiting hepatic gluconeogenesis [18,19,20].

In parallel to skeletal muscle, liver, and adipose tissue IR, impaired glucose metabolism in the brain is observed during meta-inflammation [21]. Reduced glucose metabolism severely disrupts cognitive capacity and is observed in the most common neurodegenerative diseases. In aged individuals, neurons have a reduced capacity to uptake glucose, exhibit inherent dysfunction in neuronal activities (such as less capacity to transmit signals and energy production through glycolysis and OXPHOS), and show a lack of glial help. This is likely due to both a decrease in neuronal insulin sensitivity and basal activation of microglia due to systemic inflammation incurring suboptimal glucose availability to neurons [22,23,24,25]. It is important to note that when glucose metabolism and peripheral insulin resistance are studied between young and elderly adults, the differences observed become insignificant when adjusting for increased visceral fat in elderly cohorts [24]. Many meta-inflammation-associated cytokines, independent of other stimuli, cause elevated basal microglia activation similar to what is seen in the aged brain. Microglial cells are macrophage-like innate immune cell residents in the central nervous system. Activated microglia within aged brains have severely reduced ability to protect against age-associated neurodegenerative diseases such as Alzheimer’s, Parkinson’s, dementia, and others, demonstrating the capacity of meta-inflammation to potentiate disease incidence as we age [22].

### 2.2. Inflammaging

There is likely no single incident common to every individual that initiates inflammaging, but rather genetic, lifestyle, and environmental factors compounding over time. During the aging process, hematopoietic stem cells are skewed to produce more myeloid cells and the remaining lymphoid cells have decreased functionality and an increased propensity to undergo replicative senescence [26]. Such age-related clonal expansion of hematopoietic progenitor cells not only gives rise to disruptive and recurrent genetic variants but is also directly associated with the age-related development of inflammation [27]. Reduced functionality of immune cells is a key feature of inflammaging. Lymphocytes (B and T cells) are the best-studied group due to their significant dysfunction. Their inability to provide protection yields elevated infection rates and reduced responsiveness to vaccination in the elderly [28]. Lymphocytes phenotypically have a reduced ability to respond to new antigens, but memory recall appears less impacted. It has been proposed that the higher immune cell turnover rates that occur in obesity may accelerate this immune cell dysfunction, but further validation is needed [29].

## 3. Macrophage, Meta-Inflammation, and Inflammaging

### 3.1. Origin and Distribution of Macrophages

**Figure 1:**
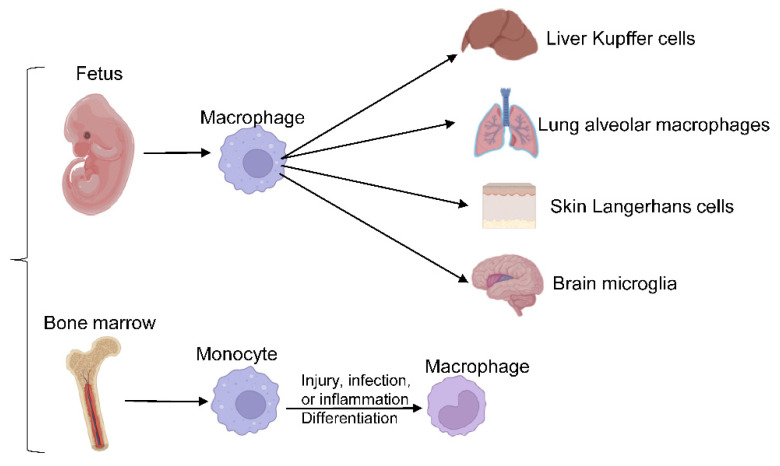
Macrophages differentiation. Embryo-derived macrophages differentiate into different tissue-resident macrophages, responding to acute or chronic demands from tissues in adulthood.

Macrophages are crucial players in innate immunity and the most widespread immune lineage in almost all tissues. They derive from either embryonic precursors or bone marrow (BM) hematopoietic stem/progenitor cells. Embryo-derived macrophages are crucial for proper tissue development and remodeling during fetal development [6]. Disruption of these populations results in growth retardation or mortality [30]. Tissue-resident macrophages, including liver Kupffer cells, skin Langerhans cells, brain microglia, lung alveolar macrophages, and others, were revealed to be of embryonic origin through elegant studies employing gate-mapping strategies in genetic mouse models. Embryo-derived macrophage populations maintain their populations in a self-sustaining manner under normal conditions [30,31,32]. In adulthood, macrophages evolve from BM-derived progenitors to maintain circulating monocyte/macrophage populations and respond to acute or chronic demands from tissues. Upon injury, infection, or sterile inflammation, monocytes are recruited and terminally differentiate into macrophages, as shown in Figure 1.

Both BM-derived and embryo-derived macrophages commonly coexist in the same tissues and work together to accomplish their functions. Various types of macrophages with unique functions and phenotypes are assembled in the gut in order to deal with the normal gut flora and oral-derived antigens [33]. Secondary lymphoid organs are also populated by various types of macrophages, such as marginal zone macrophages and sub-capsular sinus macrophages. Unique macrophages accumulate in the immune-privileged tissues of the brain, eye, and testes to perform pivotal roles in tissue remodeling and homeostasis [7,34].

### 3.2. Pathogenic Changes of Macrophages during Meta-Inflammation or Inflammaging and Related Molecular Mechanism

Depending on the stimuli or signals from the microenvironment, macrophages can quickly employ highly orchestrated signaling networks to mount appropriate responses.

We discuss the features of macrophage share in meta-inflammation and inflammaging activation in the following section.

#### 3.2.1. The Plasticity of Macrophage Activation and Relevant Molecular Mechanism

Macrophages are highly plastic to allow fast phenotypic switches for rapid responses to complex and diverse microenvironmental cues. Several models have been proposed to depict macrophage-activation features. The most popular model, macrophage polarization, characterizes macrophages into two classes: classically activated (M1) and alternatively activated (M2) macrophages [35]. This model is based on ex vivo observations of macrophage responses to T helper 1 (Th1) or Th2 stimuli and has become antiquated. Modifications to this model have sought to capture macrophages’ sophisticated actions in various tissues and physiological contexts [12,34,36]. However, no currently available model allows for comprehensive annotation of complex macrophage features under different conditions [34,37]. Our group recently created a high-resolution macrophage annotation program termed MacSpectrum [12]. MacSpectrum annotates macrophages based on their differentiation and polarization states, using two indices to capture dynamic transitions of macrophage actions under both in vitro and in vivo conditions.

Pro-inflammatory markers are highly expressed in many elderly or obese adults, even in the absence of clinically active disease [38]. For instance, macrophages will detect and respond to these pro-inflammatory mediators in both aging and obese tissues. Increased chemokine and cytokine levels were reported in the elderly and obese blood with overlapping patterns [9]. Supporting this theory, the adipose tissue macrophage (ATM) compartment in old mice is drastically different from that in young mice, with a higher percentage of M1-like, pro-inflammatory macrophages than their young counterparts, without an increase in overall macrophage number (unlike in obesity) [39]. Of note, studies using aging mice or humans are confounded by inherent visceral adipose tissue accumulation, as humans, mice, and rats advance with age. Similarly, visceral adipose tissue and systemic inflammation are hallmarks of obesity in both mouse models and human studies, characterized by increased number of macrophages, and other immune cells attribute to residential macrophage expansion and increased recruitment [9]. Moreover, the proportion of inflammation status of ATMs were drastically increased, resulting in higher levels of proinflammatory cytokine production, and systemic low-degree inflammation [40].

The mechanisms underlying macrophage polarized activation under meta-inflammation or inflammaging have been extensively investigated. Under the stress of obesity or aging, adipose tissue (AT) secretes certain adipokines/chemokines, which drive the recruitment of circulating immune cells. In addition, anti-inflammatory AT-derived adipokine/cytokine production is reduced, further exacerbating the overall AT inflammatory profile. One such anti-inflammatory adipokine is adiponectin, which is reduced in obese AT and plasma. In culture, human monocytes treated with recombinant adiponectin differentiate to an anti-inflammatory, M2-like phenotype [41,42,43]. Other than adipokine dysfunction, DAMPs and PAMPs, including LPS, interferon-γ (IFN-γ), and other TLR activators, are elevated in obese or aging individuals. These stimuli promote the M1 polarization of macrophages and activate downstream adapter proteins (e.g., MyD88), which induce the expression of pro-inflammatory genes, such as IL-1β, IL-18, and TNF-α [22]. In addition, epigenetic modifications exert an additional layer of impact on macrophage activation heterogeneity. Inhibition of histone deacetylases (HDACs) in obesity-induced diabetes models decreased body weight and blood glucose, and increased insulin sensitivity, in part, through suppressing pro-inflammatory macrophage activation and promoting alternative (M2) activation [44,45]. Furthermore, obese adults with T2D have higher levels of plasma IFN-γ, which can selectively silence the anti-inflammatory pathways by recruiting EZH2 and H3K27me3 to anti-inflammatory cytokine gene loci [46]. Interestingly, in a mouse model of aging-induced osteoporosis, similar molecular pattern shift was also observed. It was reported that increased EZH2 and decreased HDAC9 could promote age-associated osteoporosis and likely through increasing macrophage-dependent recruitment of T cells to the joints [47].

#### 3.2.2. Interplays of Macrophages with Other Immune Cells in Meta-Inflammation or Inflammaging

Macrophages, as pivotal to immune response, can interplay with other immune cells during inflammation, especially T cells. One primary aspect of macrophage function is interaction with adaptive immune cells, such as T cells, as antigen-presenting cells (APCs) to engaging adaptive immunity obesity or aging. In obese mice, macrophages with a deficiency of MHC II reduced the accumulation of effector/memory phenotype CD4+ T cells in white adipose tissue (WAT), which indicated the significance of MHCII-dependent signals from adipose tissue macrophages (ATM) in regulating T cell activation and maturation in meta-information [48]. In parallel, under inflammaging conditions such as Rheumatoid arthritis (RA), macrophages can directly regulate T cells recruitment, differentiation, and activation into RA synovium [49]. In the RA mouse model, increased infiltration of macrophages in RA synovium further attracted CXCR6+ T to the site to improve synovial inflammation [49,50]. Besides recruitment, macrophages can also promote CD4 T helper cells to differentiate to be T helper cells in RA mouse models, which was confirmed in human studies [51,52]. Moreover, macrophages can polarize CD4+ T or Th17 cells through secretion of IL-12, IL-1β, or IL-6 in RA [50,53]. It is necessary to further mechanistically investigate the interplays of macrophage and adaptive immune cells and their pathological impact on health risks associated with obesity and aging.

#### 3.2.3. Cellular Metabolism Reprogramming and Associated Mechanism

**Figure 2:**
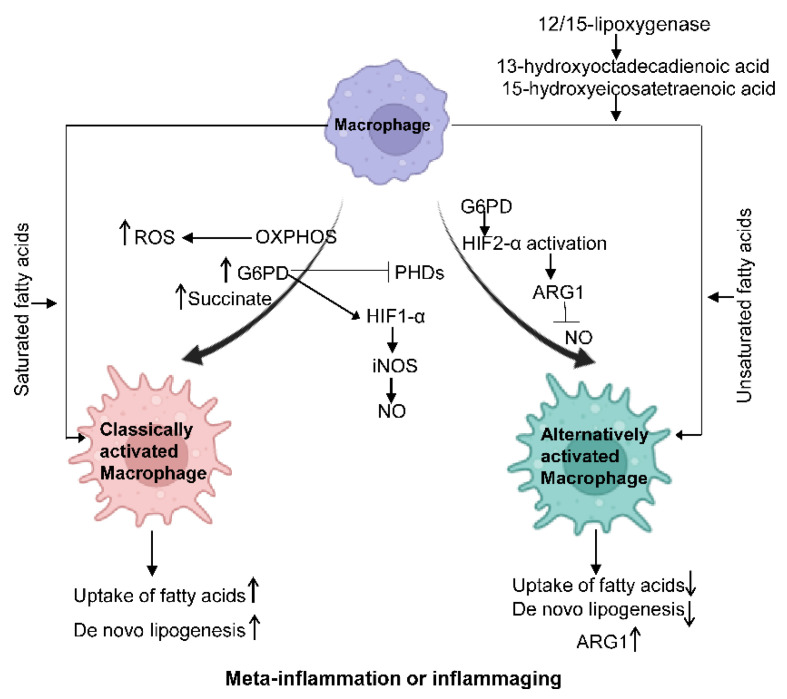
Macrophage metabolism changes under different states. Classically activated.

Metabolic reprogramming of macrophages is vital to developing and maintaining their functional phenotypes. Under meta-inflammation and inflammaging conditions, macrophages preferentially polarize towards a pro-inflammatory state (M1-like). M2-like macrophages are more abundant in young, lean individuals. Accordingly, macrophage intracellular metabolism is also reprogrammed to support such activation demands, as shown in Figure 2.

Macrophages show increased glycolysis, ROS, G6PD, and the TCA intermediate succinate. PHD expression is inhibited, and HIF1-α is activated, which further promotes the production of NO. In alternatively activated macrophages, HIF2-α is activated through ARG1 to suppress NO production. Saturated fatty acids can facilitate the polarization of classically activated macrophages, while unsaturated fatty acids can promote the polarization of alternatively activated macrophages. IL-4-induced 12/15-lipoxygenase can foster alternative macrophage activation via the generation of PPARγ ligands such as 13-hydroxyoctadecadienoic acid and 15-hydroxyeicosatetraenoic acid.

##### Glucose Metabolism

The rapid activation of macrophages demands a fast energy supply, evidenced by increased rates of glycolysis and mitochondrial oxidative phosphorylation (OXPHOS), a shared metabolic character in both aging and obesity. It should be noted that as the the OXPHOS rate increases, its by-product reactive oxygen species (ROS) increases, a major cell stressor [48]. Hexokinase activity, glucose-6-phosphate dehydrogenase (G6PD), and TCA intermediate succinate are increased in M1-polarized macrophages, which, in turn, inhibit the expression of prolyl hydroxylases (PHDs) and stabilize hypoxia-inducible factor 1-α (HIF1-α) via hydroxylation, resulting in IL-1β production in macrophages [54,55]. On the other side, HIF2-α activation primarily occurs in alternatively activated macrophages and promotes the expression of Arginase1 and suppresses NO production [51]. Further, the mechanistic target of rapamycin complex 1 (mTORC1) modulation impacts metabolic dysfunction. mTORC1 is an integral part of insulin, glucose, leptin, and growth factor signaling cascades to regulate metabolism. Defects in macrophage mTORC1 expression protect against obesity-induced AT inflammation and IR, likely due to a suppressed rate of glycolysis and caspase-1 activity, possibly inhibiting M1 polarization [56]. Additionally, suppressed mTORC1 can inhibit M1 polarization during the aging process [3,4,5,6].

##### Fatty Acid Metabolism

In macrophages, lipoprotein particles, the primary source of fatty acids, are crucial for polarized macrophage activation [53,54,55]. M1 macrophages have reduced uptake of fatty acids compared to M2 macrophages. In obesity, PPARγ ligands, such as 13-hydroxyoctadecadienoic acid and 15-hydroxyeicosatetraenoic acid, can be generated by the IL-4 inducible 12/15-lipoxygenase to foster alternative macrophage activation. In healthy adults aged over 50 years, the fatty acid profile changes following aging sabotage the switch from M1 to M2 through suppressing PPARγ activity [57]. Saturated fatty acids activate NLRP3 in primed macrophages, while unsaturated fatty acids suppress inflammasome activation [53,54,55]. Lastly, triglycerides taken up by macrophages can undergo lipolysis to generate fatty acids to support M2 activation, which is an example of cell-intrinsic lysosomal lipolysis supporting macrophage alternative function [56].

##### Amino Acid Metabolism

The role of some amino acids, including glutamine and arginine, in macrophage polarization has been extensively investigated; L-arginine metabolism is strictly controlled in macrophages through iNOS and ARG1. L-arginine can be broken down to NO and L-citrulline by iNOS or to ornithine and urea by ARG1. The expression of iNOS and the production of NO are important features of inflammatory activation of macrophages in both mouse and human studies [58,59]. Additionally, alternative activation of macrophages is characterized by highly expressed ARG1 that promotes polyamine generation, which is essential for collagen synthesis and cell proliferation [60,61].

##### Other Metabolic Changes

In addition to glucose, fatty acids, and amino acid metabolism, macrophage vitamins and iron metabolism are also altered in elderly obese individuals. Vitamin D shortage can trigger inflammation and has been linked to immune-mediated diseases such as T2D [62]. Vitamin A regulates the differentiation and function of macrophages and enhances their phagocytic capacity in mouse studies [61]. For iron metabolism reported in mouse models, inflammatory macrophages employ an iron retention program and display increased Ferritin and decreased Ferroportin phenotype; in contrast, anti-inflammatory macrophages are marked with the opposite phenotype and favor an iron-export mode [63].

## 4. Macrophages and Meta-Inflammation or Inflammaging-Related Diseases

Obesity and aging are risk factors for a plethora of diseases in humans. The combination of these two conditions could further exacerbate health risks for certain diseases, including ischemic stroke, rheumatoid arthritis (RA), osteoarthropathies (OA), and others.

### 4.1. Ischemic Stroke

Strokes rank as the third leading cause of morbidity and the second for mortality worldwide [64]. Incidence increases with aging, regardless of gender. Moreover, strokes result from the stenosis of arteries, primarily caused by atherosclerosis, which is a chronic inflammatory disease [65]. Inflammaging is believed to be a major risk factor for stroke. Clinical studies have shown that brain microglial activation can be detected as early as 24–48 h after a stroke [66]. Microglia are quickly recruited to brain lesions to clear dead neurons, mitigate local inflammation, and assist in neuron functional restoration [23,66,67]. Foam cells, as a key player in atherosclerosis, not only accumulate as fatty streaks but also release pro-inflammatory cytokines and orchestrate pathological tissue remodeling [68]. These foam cells and other specific macrophage phenotypes can impact the stability of atherosclerotic plaques in various ways [69]. However, many studies fall short in depicting a comprehensive landscape that captures the intertwined inflammation and lipid handling aspects that are unique to macrophage-derived foam cells. A lack of understanding of the complexities of atherogenesis has hindered the predictability of symptomatic and asymptomatic outcomes in patients. Future studies to define the molecular mechanisms connecting lipid metabolism and macrophage activation will facilitate the development of next-generation atherosclerosis therapeutic strategies.

### 4.2. RA and OA

RA is an autoimmune disease characterized by persistent synovitis, systemic inflammation, cachexia, and the destruction of joints [70]. Cachexia describes a “wasting” state where patients experience extreme loss of adipose tissue and muscle. In aged individuals, muscle deterioration and loss of muscle function are typical; coupled with obesity, muscle wasting is known as “sarcopenic obesity” and contributes significantly to the disability and mortality of elderly individuals [71,72]. Obesity not only increases the risk of developing RA but also reduces the efficacy of RA treatment [73]. So, both meta-inflammation and inflammaging promote the development of RA. Macrophages can recruit T cells to sabotage the structure of joints. Joint destruction advances with the infiltration of synovial macrophages. In RA mouse models, removal of all macrophages reduced inflammation and joint destruction, which suppressed the progression of RA [74]. Similar to RA, osteoarthropathies (OAs) are characterized by joint destruction caused by immune microenvironment malfunction and are tightly associated with aging and obesity. Synovial play pivotal roles in the early stage of OA. Inflammatory macrophages can interfere with matrix production by increasing the generation of proteolytic enzymes, such as matrix metalloproteinase and cyclooxygenase, damaging cartilage, and promoting OA [75,76]. It is believed that alternatively activated macrophages, which are known to be disfavored in meta-inflammation and inflammaging, are crucial for the repair of tissues such as articular cartilage during OA treatment [77].

### 4.3. Sarcopenia

Sarcopenia is defined as skeletal muscle disorder, characterized by loss of muscle mass and function, which occurs in older people for the imbalance of muscle protein anabolic and catabolic following aging [78]. It was reported that inflammation can promote the development of sarcopenia [79]. Moreover, sarcopenia can be worsened by obese conditions, termed as sarcopenic obesity (SOB). Recently investigation in a co-culture system using mouse cells revealed that M1 macrophages could promote the proliferation of myogenic precursor cells (MPC), whereas M2 macrophages could promote the differentiation of MPCs, suggesting a potential SOB mitigation strategy by targeting macrophage activation status [80]. Macrophages can also affect the remodeling of muscle through secretion of different cytokines, such as IL6, and IL10. IL10 was reported to promote the phenotypic transition of macrophages to increase muscle fiber repair [81], and IL6 favors muscle regeneration in mice [82]. Furthermore, extracellular matrix components are necessary in efficient muscle repair, which can be promoted by macrophages through the secretion of MMP-14 in mice [83].

## 5. Summary

The average life expectancy is rising and the impact of obesity on the health care system has become a worldwide concern. In addition, changes in socioeconomics, food availability, climate, and lifestyle habits have conferred unprecedented levels of overweight and obese individuals in most countries. Aging and obesity are independently becoming forceful destroyers of human health and quality of life. Moreover, comorbid obesity and advanced age are an even greater threat to public health. Both conditions strongly promote inflammation, which makes immune cells promising therapeutic targets. Macrophages, in particular, are of great interest in both aging- and obesity-associated health risks. Dynamic activation states of macrophages empower them to be central to innate immunity against pathogens and essential for normal tissue function homeostasis. Plasticity, a crucial feature of macrophage activation, is achieved by tightly orchestrated intracellular molecular networks and dynamically adjusted communications to other cells and the extracellular matrix. It is necessary to consider diverse factors that are significantly altered under obese and aging conditions to understand the action of macrophages, and harness their potent functions to mitigate meta-inflammation, inflammaging, and their associated health risks.

The factors that affect macrophages’ plasticity are complicated. These factors can be studied at both the macroscopical and microcosmic levels. The macroscopical factors refer to stimuli from the macrophages’ surrounding environment, including other cells (cytokines, etc.), extrinsic factors (DAMPs, PAMPs, etc.), and metabolites. Microcosmic factors include intracellular metabolism of glucose, proteins, fatty acids, and others. The regulation of macrophages is subtle and complicated. In this review, we focused on activation states and their connections to various obesity- and aging-associated diseases. More efforts into understanding the etiology and pathology of each disease are needed to discover effective treatments, exemplified by the nuanced functions of macrophages in each case. With the advancement of systems approaches, such as single cell RNA sequencing (scRNA-seq), cellular indexing of transcriptomes and epitopes by sequencing (CITE-seq), and assay for transposase-accessible chromatin using sequencing (ATAC-seq), we are able to resolve mysteries about macrophages and their roles in diseases. For example, the MacSpectrum program that was generated by our lab based on scRNA-seq data allows for the capture of dynamic macrophage states in vitro and in vivo, providing unique genomic information about macrophages [12]. The generation of MacSpectrum and other programs alike will help us grow into a new era of understanding macrophages [84]. Stimulation-specific gene sets should be cross-analyzed with different disease and tissue contexts, so in the future, we can use the MacSpectrum roadmap to depict macrophages’ features under complex conditions, like aging and obesity. More research like MacSpectrum will provide a better understanding of the nuances of macrophages and will expel the confounding role of macrophages in disease.

## References

[1] The Department of Economic and Social Affairs of the United Nations (2017). World Population Ageing 2017-Hights (ST/ESA/SER.A/397).

[2] Crunkhorn S. (2020). Reversing inflammaging. Nat. Rev. Drug Discov..

[3] GBD 2017 Risk Factor Collaborators (2018). Global, regional, and national comparative risk assessment of 84 behavioural, environmental and occupational, and metabolic risks or clusters of risks for 195 countries and territories, 1990–2017: A systematic analysis for the Global Burden of Disease Study 2017. Lancet.

[4] Qu L.L., Yu B., Li Z., Jiang W.X., Jiang J.D., Kong W.J. (2016). Gastrodin Ameliorates Oxidative Stress and Proinflammatory Response in Nonalcoholic Fatty Liver Disease through the AMPK/Nrf2 Pathway. Phytother. Res..

[5] Wynn T.A., Vannella K.M. (2016). Macrophages in Tissue Repair, Regeneration, and Fibrosis. Immunity.

[6] Varol C., Mildner A., Jung S. (2015). Macrophages: Development and tissue specialization. Annu. Rev. Immunol..

[7] Ginhoux F., Jung S. (2014). Monocytes and macrophages: Developmental pathways and tissue homeostasis. Nat. Rev. Immunol..

[8] Li C., Xu M.M., Wang K., Adler A.J., Vella A.T., Zhou B. (2018). Macrophage polarization and meta-inflammation. Transl. Res..

[9] Reyes-Farias M., Fos-Domenech J., Serra D., Herrero L., Sanchez-Infantes D. (2021). White adipose tissue dysfunction in obesity and aging. Biochem. Pharmacol..

[10] Yan L.L., Daviglus M.L., Liu K., Stamler J., Wang R., Pirzada A., Garside D.B., Dyer A.R., Van Horn L., Liao Y. (2006). Midlife body mass index and hospitalization and mortality in older age. JAMA.

[11] Mouton A.J., Li X., Hall M.E., Hall J.E. (2020). Obesity, Hypertension, and Cardiac Dysfunction: Novel Roles of Immunometabolism in Macrophage Activation and Inflammation. Circ. Res..

[12] Li C., Menoret A., Farragher C., Ouyang Z., Bonin C., Holvoet P., Vella A.T., Zhou B. (2019). Single cell transcriptomics based-MacSpectrum reveals novel macrophage activation signatures in diseases. JCI Insight.

[13] Oishi Y., Manabe I. (2016). Macrophages in age-related chronic inflammatory diseases. NPJ Aging Mech. Dis..

[14] Koenen M., Hill M.A., Cohen P., Sowers J.R. (2021). Obesity, Adipose Tissue and Vascular Dysfunction. Circ. Res..

[15] Li C., Qu L., Farragher C., Vella A., Zhou B. (2019). MicroRNA Regulated Macrophage Activation in Obesity. J. Transl. Int. Med..

[16] Jounai N., Kobiyama K., Takeshita F., Ishii K.J. (2012). Recognition of damage-associated molecular patterns related to nucleic acids during inflammation and vaccination. Front. Cell Infect. Microbiol..

[17] Matz A., Qu L., Karlinsey K., Zhou B. (2022). Impact of microRNA Regulated Macrophage Actions on Adipose Tissue Function in Obesity. Cells.

[18] Hotamisligil G.S. (2017). Inflammation, metaflammation and immunometabolic disorders. Nature.

[19] Lee Y.S., Olefsky J. (2021). Chronic tissue inflammation and metabolic disease. Genes Dev..

[20] Arfianti A., Pok S., Barn V., Haigh W.G., Yeh M.M., Ioannou G.N., Teoh N.C., Farrell G.C. (2020). Exercise retards hepatocarcinogenesis in obese mice independently of weight control. J. Hepatol..

[21] Samuel V.T., Petersen K.F., Shulman G.I. (2010). Lipid-induced insulin resistance: Unravelling the mechanism. Lancet.

[22] Madore C., Yin Z., Leibowitz J., Butovsky O. (2020). Microglia, Lifestyle Stress, and Neurodegeneration. Immunity.

[23] Kofler J., Wiley C.A. (2011). Microglia: Key innate immune cells of the brain. Toxicol. Pathol..

[24] Spielman L.J., Little J.P., Klegeris A. (2014). Inflammation and insulin/IGF-1 resistance as the possible link between obesity and neurodegeneration. J. Neuroimmunol..

[25] van den Beld A.W., Kaufman J.M., Zillikens M.C., Lamberts S.W.J., Egan J.M., van der Lely A.J. (2018). The physiology of endocrine systems with ageing. Lancet Diabetes Endocrinol..

[26] Sera Y., Nakata Y., Ueda T., Yamasaki N., Koide S., Kobayashi H., Ikeda K.I., Kobatake K., Iwasaki M., Oda H. (2021). UTX maintains the functional integrity of the murine hematopoietic system by globally regulating aging-associated genes. Blood.

[27] Shlush L.I. (2018). Age-related clonal hematopoiesis. Blood.

[28] Varricchi G., Bencivenga L., Poto R., Pecoraro A., Shamji M.H., Rengo G. (2020). The emerging role of T follicular helper (TFH) cells in aging: Influence on the immune frailty. Ageing Res. Rev..

[29] Ying W., Fu W., Lee Y.S., Olefsky J.M. (2020). The role of macrophages in obesity-associated islet inflammation and beta-cell abnormalities. Nat. Rev. Endocrinol..

[30] Yona S., Kim K.W., Wolf Y., Mildner A., Varol D., Breker M., Strauss-Ayali D., Viukov S., Guilliams M., Misharin A. (2013). Fate mapping reveals origins and dynamics of monocytes and tissue macrophages under homeostasis. Immunity.

[31] Hashimoto D., Chow A., Noizat C., Teo P., Beasley M.B., Leboeuf M., Becker C.D., See P., Price J., Lucas D. (2013). Tissue-resident macrophages self-maintain locally throughout adult life with minimal contribution from circulating monocytes. Immunity.

[32] Schulz C., Gomez Perdiguero E., Chorro L., Szabo-Rogers H., Cagnard N., Kierdorf K., Prinz M., Wu B., Jacobsen S.E., Pollard J.W. (2012). A lineage of myeloid cells independent of Myb and hematopoietic stem cells. Science.

[33] Davis M.J., Tsang T.M., Qiu Y., Dayrit J.K., Freij J.B., Huffnagle G.B., Olszewski M.A. (2013). Macrophage M1/M2 polarization dynamically adapts to changes in cytokine microenvironments in Cryptococcus neoformans infection. mBio.

[34] Das A., Sinha M., Datta S., Abas M., Chaffee S., Sen C.K., Roy S. (2015). Monocyte and macrophage plasticity in tissue repair and regeneration. Am. J. Pathol..

[35] Tilg H., Hotamisligil G.S. (2006). Nonalcoholic fatty liver disease: Cytokine-adipokine interplay and regulation of insulin resistance. Gastroenterology.

[36] Murray P.J., Allen J.E., Biswas S.K., Fisher E.A., Gilroy D.W., Goerdt S., Gordon S., Hamilton J.A., Ivashkiv L.B., Lawrence T. (2014). Macrophage activation and polarization: Nomenclature and experimental guidelines. Immunity.

[37] Biswas S.K., Mantovani A. (2010). Macrophage plasticity and interaction with lymphocyte subsets: Cancer as a paradigm. Nat. Immunol..

[38] Ferrucci L., Semba R.D., Guralnik J.M., Ershler W.B., Bandinelli S., Patel K.V., Sun K., Woodman R.C., Andrews N.C., Cotter R.J. (2010). Proinflammatory state, hepcidin, and anemia in older persons. Blood.

[39] Lumeng C.N., Liu J., Geletka L., Delaney C., Delproposto J., Desai A., Oatmen K., Martinez-Santibanez G., Julius A., Garg S. (2011). Aging is associated with an increase in T cells and inflammatory macrophages in visceral adipose tissue. J. Immunol..

[40] Lumeng C.N., DelProposto J.B., Westcott D.J., Saltiel A.R. (2008). Phenotypic switching of adipose tissue macrophages with obesity is generated by spatiotemporal differences in macrophage subtypes. Diabetes.

[41] Ohashi K., Parker J.L., Ouchi N., Higuchi A., Vita J.A., Gokce N., Pedersen A.A., Kalthoff C., Tullin S., Sams A. (2010). Adiponectin promotes macrophage polarization toward an anti-inflammatory phenotype. J. Biol. Chem..

[42] Lovren F., Pan Y., Quan A., Szmitko P.E., Singh K.K., Shukla P.C., Gupta M., Chan L., Al-Omran M., Teoh H. (2010). Adiponectin primes human monocytes into alternative anti-inflammatory M2 macrophages. Am. J. Physiol. Heart Circ. Physiol..

[43] Mandal P., Park P.H., McMullen M.R., Pratt B.T., Nagy L.E. (2010). The anti-inflammatory effects of adiponectin are mediated via a heme oxygenase-1-dependent pathway in rat Kupffer cells. Hepatology.

[44] Brown J.D., Lin C.Y., Duan Q., Griffin G., Federation A., Paranal R.M., Bair S., Newton G., Lichtman A., Kung A. (2014). NF-kappaB directs dynamic super enhancer formation in inflammation and atherogenesis. Mol. Cell.

[45] Foster S.L., Hargreaves D.C., Medzhitov R. (2007). Gene-specific control of inflammation by TLR-induced chromatin modifications. Nature.

[46] Kang K., Park S.H., Chen J., Qiao Y., Giannopoulou E., Berg K., Hanidu A., Li J., Nabozny G., Kang K. (2017). Interferon-gamma Represses M2 Gene Expression in Human Macrophages by Disassembling Enhancers Bound by the Transcription Factor MAF. Immunity.

[47] Chen Y.H., Chung C.C., Liu Y.C., Yeh S.P., Hsu J.L., Hung M.C., Su H.L., Li L.Y. (2016). Enhancer of Zeste Homolog 2 and Histone Deacetylase 9c Regulate Age-Dependent Mesenchymal Stem Cell Differentiation into Osteoblasts and Adipocytes. Stem Cells.

[48] Cho K.W., Morris D.L., DelProposto J.L., Geletka L., Zamarron B., Martinez-Santibanez G., Meyer K.A., Singer K., O’Rourke R.W., Lumeng C.N. (2014). An MHC II-dependent activation loop between adipose tissue macrophages and CD4+ T cells controls obesity-induced inflammation. Cell Rep..

[49] Kim C.H., Rott L., Kunkel E.J., Genovese M.C., Andrew D.P., Wu L., Butcher E.C. (2001). Rules of chemokine receptor association with T cell polarization in vivo. J. Clin. Investig..

[50] Stamp L.K., Easson A., Pettersson L., Highton J., Hessian P.A. (2009). Monocyte derived interleukin (IL)-23 is an important determinant of synovial IL-17A expression in rheumatoid arthritis. J. Rheumatol..

[51] Evans H.G., Gullick N.J., Kelly S., Pitzalis C., Lord G.M., Kirkham B.W., Taams L.S. (2009). In vivo activated monocytes from the site of inflammation in humans specifically promote Th17 responses. Proc. Natl. Acad. Sci. USA.

[52] Yoon B.R., Yoo S.J., Choi Y., Chung Y.H., Kim J., Yoo I.S., Kang S.W., Lee W.W. (2014). Functional phenotype of synovial monocytes modulating inflammatory T-cell responses in rheumatoid arthritis (RA). PLoS ONE.

[53] Pene J., Chevalier S., Preisser L., Venereau E., Guilleux M.H., Ghannam S., Moles J.P., Danger Y., Ravon E., Lesaux S. (2008). Chronically inflamed human tissues are infiltrated by highly differentiated Th17 lymphocytes. J. Immunol..

[54] Newsholme P., Curi R., Gordon S., Newsholme E.A. (1986). Metabolism of glucose, glutamine, long-chain fatty acids and ketone bodies by murine macrophages. Biochem. J..

[55] Tannahill G.M., Curtis A.M., Adamik J., Palsson-McDermott E.M., McGettrick A.F., Goel G., Frezza C., Bernard N.J., Kelly B., Foley N.H. (2013). Succinate is an inflammatory signal that induces IL-1beta through HIF-1alpha. Nature.

[56] Jiang H., Westerterp M., Wang C., Zhu Y., Ai D. (2014). Macrophage mTORC1 disruption reduces inflammation and insulin resistance in obese mice. Diabetologia.

[57] Pararasa C., Ikwuobe J., Shigdar S., Boukouvalas A., Nabney I.T., Brown J.E., Devitt A., Bailey C.J., Bennett S.J., Griffiths H.R. (2016). Age-associated changes in long-chain fatty acid profile during healthy aging promote pro-inflammatory monocyte polarization via PPARgamma. Aging Cell.

[58] Orecchioni M., Ghosheh Y., Pramod A.B., Ley K. (2019). Macrophage Polarization: Different Gene Signatures in M1(LPS+) vs. Classically and M2(LPS-) vs. Alternatively Activated Macrophages. Front. Immunol..

[59] Sun C., Sun L., Ma H., Peng J., Zhen Y., Duan K., Liu G., Ding W., Zhao Y. (2012). The phenotype and functional alterations of macrophages in mice with hyperglycemia for long term. J. Cell Physiol..

[60] Fuentes E., Fuentes F., Vilahur G., Badimon L., Palomo I. (2013). Mechanisms of chronic state of inflammation as mediators that link obese adipose tissue and metabolic syndrome. Mediat. Inflamm..

[61] Van Dyken S.J., Locksley R.M. (2013). Interleukin-4- and interleukin-13-mediated alternatively activated macrophages: Roles in homeostasis and disease. Annu. Rev. Immunol..

[62] Illescas-Montes R., Melguizo-Rodriguez L., Ruiz C., Costela-Ruiz V.J. (2019). Vitamin D and autoimmune diseases. Life Sci..

[63] Winn N.C., Volk K.M., Hasty A.H. (2020). Regulation of tissue iron homeostasis: The macrophage “ferrostat”. JCI Insight.

[64] Lozano R., Naghavi M., Foreman K., Lim S., Shibuya K., Aboyans V., Abraham J., Adair T., Aggarwal R., Ahn S.Y. (2012). Global and regional mortality from 235 causes of death for 20 age groups in 1990 and 2010: A systematic analysis for the Global Burden of Disease Study 2010. Lancet.

[65] Li C., Qu L., Matz A.J., Murphy P.A., Liu Y., Manichaikul A.W., Aguiar D., Rich S.S., Herrington D.M., Vu D. (2022). AtheroSpectrum Reveals Novel Macrophage Foam Cell Gene Signatures Associated With Atherosclerotic Cardiovascular Disease Risk. Circulation.

[66] Price C.J., Wang D., Menon D.K., Guadagno J.V., Cleij M., Fryer T., Aigbirhio F., Baron J.C., Warburton E.A. (2006). Intrinsic activated microglia map to the peri-infarct zone in the subacute phase of ischemic stroke. Stroke.

[67] Xiong X.Y., Liu L., Yang Q.W. (2016). Functions and mechanisms of microglia/macrophages in neuroinflammation and neurogenesis after stroke. Prog. Neurobiol..

[68] Moore K.J., Sheedy F.J., Fisher E.A. (2013). Macrophages in atherosclerosis: A dynamic balance. Nat. Rev. Immunol..

[69] Newby A.C., George S.J., Ismail Y., Johnson J.L., Sala-Newby G.B., Thomas A.C. (2009). Vulnerable atherosclerotic plaque metalloproteinases and foam cell phenotypes. Thromb. Haemost..

[70] Smolen J.S., Aletaha D., McInnes I.B. (2016). Rheumatoid arthritis. Lancet.

[71] Roubenoff R. (2009). Rheumatoid cachexia: A complication of rheumatoid arthritis moves into the 21st century. Arthritis Res. Ther..

[72] Rall L.C., Roubenoff R. (2004). Rheumatoid cachexia: Metabolic abnormalities, mechanisms and interventions. Rheumatology.

[73] Iannone F., Lopalco G., Rigante D., Orlando I., Cantarini L., Lapadula G. (2016). Impact of obesity on the clinical outcome of rheumatologic patients in biotherapy. Autoimmun. Rev..

[74] Udalova I.A., Mantovani A., Feldmann M. (2016). Macrophage heterogeneity in the context of rheumatoid arthritis. Nat. Rev. Rheumatol..

[75] Haltmayer E., Ribitsch I., Gabner S., Rosser J., Gueltekin S., Peham J., Giese U., Dolezal M., Egerbacher M., Jenner F. (2019). Co-culture of osteochondral explants and synovial membrane as in vitro model for osteoarthritis. PLoS ONE.

[76] Manferdini C., Paolella F., Gabusi E., Silvestri Y., Gambari L., Cattini L., Filardo G., Fleury-Cappellesso S., Lisignoli G. (2016). From osteoarthritic synovium to synovial-derived cells characterization: Synovial macrophages are key effector cells. Arthritis Res. Ther..

[77] Dai M., Sui B., Xue Y., Liu X., Sun J. (2018). Cartilage repair in degenerative osteoarthritis mediated by squid type II collagen via immunomodulating activation of M2 macrophages, inhibiting apoptosis and hypertrophy of chondrocytes. Biomaterials.

[78] Cruz-Jentoft A.J., Sayer A.A. (2019). Sarcopenia. Lancet.

[79] Beyer I., Mets T., Bautmans I. (2012). Chronic low-grade inflammation and age-related sarcopenia. Curr. Opin. Clin. Nutr. Metab. Care.

[80] Arnold L., Henry A., Poron F., Baba-Amer Y., van Rooijen N., Plonquet A., Gherardi R.K., Chazaud B. (2007). Inflammatory monocytes recruited after skeletal muscle injury switch into antiinflammatory macrophages to support myogenesis. J. Exp. Med..

[81] Deng B., Wehling-Henricks M., Villalta S.A., Wang Y., Tidball J.G. (2012). IL-10 triggers changes in macrophage phenotype that promote muscle growth and regeneration. J. Immunol..

[82] Zhang J., Muri J., Fitzgerald G., Gorski T., Gianni-Barrera R., Masschelein E., D’Hulst G., Gilardoni P., Turiel G., Fan Z. (2020). Endothelial Lactate Controls Muscle Regeneration from Ischemia by Inducing M2-like Macrophage Polarization. Cell Metab..

[83] Peck B.D., Murach K.A., Walton R.G., Simmons A.J., Long D.E., Kosmac K., Dungan C.M., Kern P.A., Bamman M.M., Peterson C.A. (2022). A muscle cell-macrophage axis involving matrix metalloproteinase 14 facilitates extracellular matrix remodeling with mechanical loading. FASEB J..

[84] Karlinsey K., Qu L., Matz A.J., Zhou B. (2022). A novel strategy to dissect multifaceted macrophage function in human diseases. J Leukoc. Biol..

